# Reconstruction of iliac crest defect after autogenous harvest with bone cement and screws reduces donor site pain

**DOI:** 10.1186/s12891-018-2167-7

**Published:** 2018-07-19

**Authors:** Jing Zhang, Yuxuan Wei, Yue Gong, Yang Dong, Zhichang Zhang

**Affiliations:** 1 Department of Orthopaedic Surgery, Shanghai Jiao Tong University Affiliated Sixth People’s Hospital, Shanghai, 200233 China; 2 Department of Breast Surgery, Key Laboratory of Breast Cancer in Shanghai, Fudan University Shanghai Cancer Center, Fudan University, Shanghai, 200032 China

**Keywords:** Iliac crest bone graft, Morbidity, Reconstruction of donor site, Bone cement

## Abstract

**Background:**

The iliac crest is the most common autogenous bone graft donor site, although associated with postoperative pain, functional disability, cosmesis, morphology and surgical satisfaction. We assessed each aspect above by comparing iliac crest reconstruction with bone cement and screws following harvest with no reconstruction.

**Methods:**

We evaluated patients who underwent large iliac crest harvesting, including ten patients who underwent iliac crest defect reconstruction with bone cement and cancellous screws (R group) and ten randomly matched patients without reconstruction (NR group) were evaluated prospectively in the same period. Local pain, cosmesis and other complications were assessed postoperatively at 1 week, 6 weeks, 3 months and 6 months.

**Results:**

Pain, cosmesis and satisfaction of patients significantly differed between the two groups. The R group exhibited less complications and lower pain visual analogue scores at postoperative 1 week (*p* < 0.001), 6 weeks (*p* < 0.001) and 3 months (*p* < 0.01) but not at 6 months, at which time patients reported almost no pain. One patient reported pain for more than 1 year in the NR group. The R group exhibited better cosmesis, morphology and satisfaction than the NR group. In the NR group, one patient suffered pain when sitting up and another when wearing a belt.

**Conclusion:**

Postoperative pain can be reduced and cosmesis can be improved through reconstructing the iliac crest defects after autogenous harvesting with bone cement and cancellous screws. The technique is simple, safe and easy to implement.

## Background

Autogenous bone grafts are widely used in clinical orthopedics due to their biological and nonimmunologic properties compared with other materials. The iliac crest is the most common donor site because of its easy access, low morbidity and ability to provide sufficient quantities of both cortical and cancellous bone [1]. However, complication rates following iliac crest bone harvesting have been reported from 2 to 49%. Most patients suffer from pain which has an effect on sleep within 1 month after surgery, and even 13 to 20% of the patients experience chronic pain [2, 3]. The most common complication is pain at the donor site after that less frequently complications including secondary fracture, superficial numbness, infection, abdominal hernia and gait abnormality [4–6]. In addition, harvesting a large graft from iliac crest bone leads to some problems such as depression of surgical area, poor cosmetic appearance, having influence on walking, recreation, household chores and so on [2].

Some studies have indicated that reconstruction of iliac crest defects after harvesting can reduce complications [7–10]. Various implantation materials and techniques have been used to rebuild iliac defects. Burton and colleagues [11] reconstructed the iliac crest with a hydroxyapatite-calcium triphosphate biphasic compound, which improved the body’s ability to reform new bone but did not alleviate the pain. Furthermore, the technique is only suitable for harvesting parts of cancellous bones. Other studies have been carried out for the repair of iliac crest defects using autogenous bone [7], bovine cancellous grafts [9], polymethyl methacrylate bone cement [12] and allografts fixed with cannulated screws [10]. Although such studies provided some options to reduce the donor site pain, we still try to find a more simple and effective method.

In this study, we used a new technique with bone cement and screws to repair large iliac bicortical bone defects after autogenous harvesting for bone tumors, such as giant cell tumors of bone or other benign tumors. The purpose of this study was to evaluate the benefits of this new method.

## Methods

### Study population

Patients who underwent autogenous iliac crest grafting to repair a bone defect or nonunion after primary bone tumor excision in 2016 were selected for inclusion in this trial. The following inclusion criteria were utilized for patient selection: surgical treatment with a large iliac crest graft for repair of a bone defect or nonunion association with primary bone tumor excision; harvest of more than 40 mm × 30 mm of iliac crest; and patients aged between 18 and 60 years old. Patients with osteoporosis or who could not tolerate the surgery for iliac crest reconstruction were not included. That patients were asked whether they wanted to receive conventional procedures or the new method prior the study took place and that the control group was matched after the study population for the new method was gathered.

Twenty patients were included in this study. Ten patients who conducted reconstruction were allocated to the reconstructed group (R group) and were compared with 10 patients whose iliac crest defect was not reconstructed (NR group). All surgical procedures were performed by the same chief surgeon.

### Operation technique

**Fig. 1:**
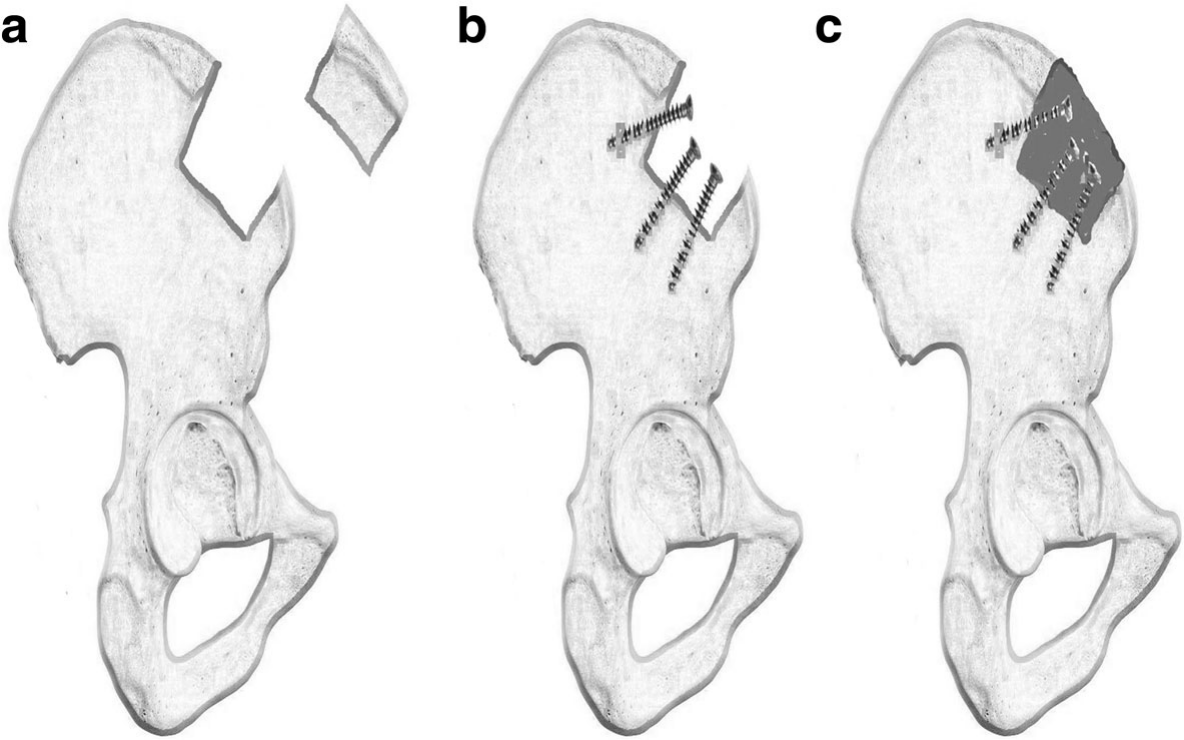
The procedure of reconstruction of the iliac crest after autografting. **a** the defect after the iliac crest harvesting. **b** Cancellous screws were implanted leaving 2—3 cm of head out of the bone as an anchor. **c** Filled bone cement into the defect and shaped them into the shape of the iliac crest contour making ensure that the cement did not extend beyond the original shape

Both groups underwent the same procedure of the harvest. The volume of the bone graft depended on the volume of the tumor. The iliac crest graft harvest was on the same side as the bone tumor. In supine position, a skin incision was made along iliac crest contour. We dissected soft tissue and harvested the required amount of iliac crest using an osteotome from 3 cm posterior to the anterior superior iliac spine (Fig. 1a). In the R group, according to the harvesting size, three or more cancellous screws were implanted leaving 2—3 cm of head end out of the bone as an anchor providing support and adhesion for the bone cement (Fig. 1b). The defect area was filled with bone cement which was formed into the shape of the iliac crest contour (Fig. 1c). Careful attention was paid to ensure that the cement did not extend beyond the original shape of the bone to avoid discomfort. After the bone cement had solidified, a drainage tube was placed, and the incision was closed. The only difference between the two groups was that the defect of the iliac crest was reconstructed in the R group, whereas no reconstruction was performed in the NR group.

### Assessment

Postoperative pain was evaluated using the pain visual analog scale (pain VAS) in both groups at 1 week, 6 weeks, 3 months and 6 months after the surgery. Functional disability was evaluated based on the pain of the patients reported when they performed 3 daily activities: sitting up, wearing a belt, and sleeping on the operation side. Cosmesis of the operated site, morphology of the defect, and surgical satisfaction scale (SSS) were only assessed at 6 months (Table 1). Complications were recorded during the follow-up and a radiologic evaluation was performed using posterior-anterior radiograph images of the pelvis at 1 week, 6 weeks, 3 months and 6 months after the surgery.

**Table 1:** The clinical assessment contents and criteria

Pain VAS	Range of 0 to 10(0 indicates no pain, 10 indicates the worst pain imaginable)
Function disability	Sitting up
	Wearing a belt
	Sleep on the operation side
Cosmesis of the operated side	Range of 0 to 10 (10 indicates that the appearance is almost no different from the contralateral side. 0 means very bad)
Depression of the defect	The same as the contralateral side (depression < 1 cm, normal)
	Depressed compared with the contralateral side(> 1 cm, depression)
Surgical satisfaction scale	Range of 0 to 10(10 indicates it’ s very satisfied. 0 indicates it’ s not satisfied at all)

Differences between groups were analyzed using Student’s t-test and Fisher’s exact test. All statistical analyses were performed using SPSS 20.0 software (SPSS Inc., Chicago, IL). *P* < 0.05 was considered as statistically significant.

## Results

**Fig. 2:**
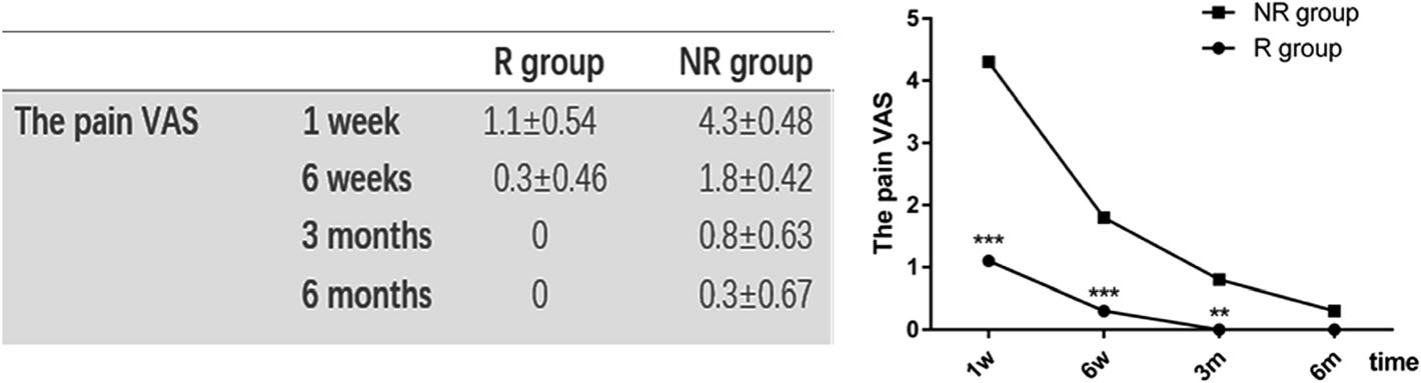
The pain VAS scores between the two groups during the postoperative 6 months. The pain VAS between the R group and the NR group was 1.1 ± 0.53 vs 4.3 ± 0.48, 0.3 ± 0.46 vs 1.8 ± 0.42, 0 vs 0.8 ± 0.63 and 0 vs 0.3 ± 0.67 at 1 week, 6 weeks, 3 months and 6 months after surgery. ** *p* < 0.01, *** *p* < 0.001

The demographic data between the two groups had no significant differences, as presented in Table 2. The pain VAS of the R group was 1.1 ± 0.53, markedly lower than that of the NR group of 4.3 ± 0.48 (*p* < 0.001) at 1 week after surgery. Similarly, the VAS score showed significant differences between the two groups at 6 weeks (0.3 ± 0.46 vs 1.8 ± 0.42, *p* < 0.001) and 3 months (0 vs 0.8 ± 0.63, *p* < 0.01). In comparison to the R group where no patient suffered from pain after 6 months (*p* = 0.29, Fig. 2). However, one patient suffered from pain for more than 1 year in the NR group.

**Table 2:** The demographic data of all patients

	R group	NR group	*P* values
Number of patients	10	10	
Age(years)	38.7 ± 14.2(18–59)	36 ± 10.2(24–53)	*p* = 0.634
Sex (F:M)	5:5	6:4	*P* = 1
Average size of iliac crest defect(cm^2^)	14.55 ± 2.0	13.5 ± 1.6	*p* = 0.297

Complications in the R group included one patient who reported thigh numbness and one who reported slight pain when wearing a waist belt during the first 6 months postoperatively, which were alleviated by the sixth month. On the other hand, two patients reported numbness on the outside of the thigh in the NR group. One patient suffered pain when wearing a waist belt and another patient reported ache when sitting up.

**Fig. 3:**
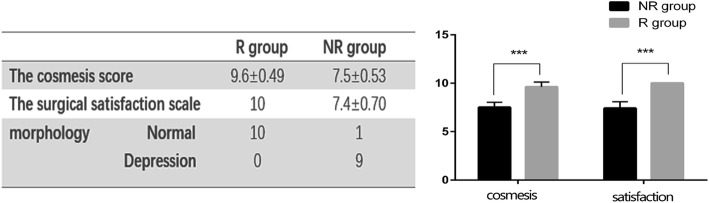
Cosmesis and surgical satisfaction scale scores between the two groups at postoperative 6 months. The cosmesis score between R group and NR group was 9.6 ± 0.49 vs 7.5 ± 0.53 and the SSS was 10 vs 7.4 ± 0.70. *** *p* < 0.001

There was a significant difference between the two groups in cosmesis assessment. The mean score of cosmesis was 9.6 in the R group, which was significantly higher than that of the NR group of 7.5 (*p* < 0.001). The result of SSS was consistent with the cosmesis score (Fig. 3). The morphology of the two groups was also significantly different. In the R group, there was no depression, whereas 9 cases of depression appeared in the NR group (*p* < 0.001, Table 2).

**Fig. 4:**
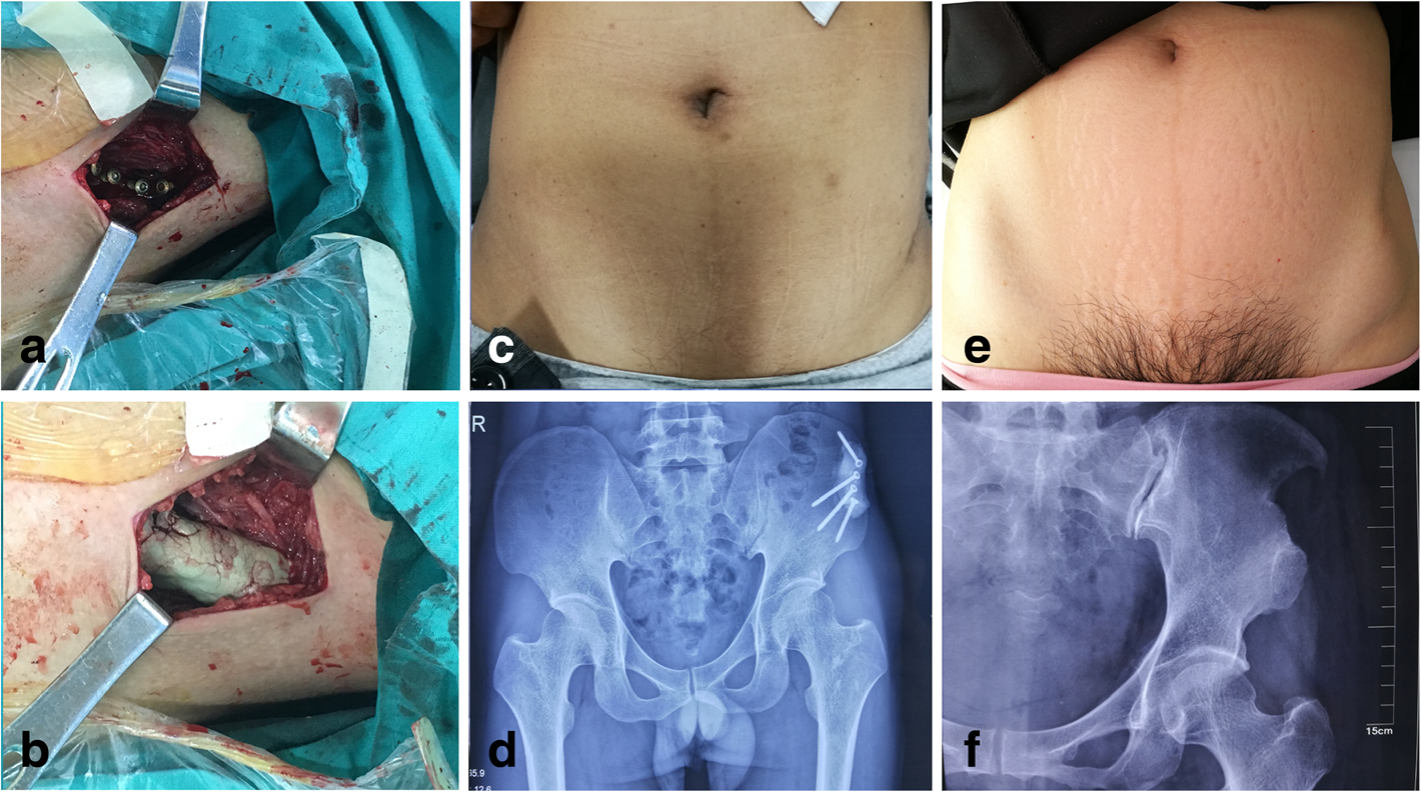
A 40 years old male (**a, b, c, d**) in R group and a 47 years old female (**e, f**) in NR group, who were diagnosed with giant cell tumor of distal femur, experienced curettage of bone tumor and iliac crest graft. **a** The screws are implanted after iliac crest harvesting. **b** Bone cement is filled into the defect area. **c** The cosmesis and morphology of the surgical area compared with contralateral. **d** posterior-anterior radiograph images of the pelvis at 6 months after surgical reconstruction. **e** and **f**, The cosmesis of the surgical area compared with contralateral and posterior-anterior radiograph images of the pelvis at 1 year after surgery without reconstruction

Posterior-anterior radiograph images of the pelvis were assessed postoperatively during the 6 months. There was no secondary fracture of bone or dislocation or fractured bone cement in both groups (Fig. 4).

## Discussion

The iliac crest remains the most frequently site for autogenous grafts with many advantages compared with other sites such as distal radius and artificial bone. However, postoperative pain and poor cosmesis are major deterrents in harvesting bone from the iliac crest. Different methods have been used to solve these problems, such as injection of local anesthetic which have achieved different levels of success [1, 13]. However, the results were still not satisfying because of the problems mentioned above. Therefore, some researchers have attempted to reconstruct the iliac crest after harvesting bone and discovered that it could alleviate donor site pain and access good cosmesis [8, 10, 14, 15].

In our study, we used bone cement and cancellous screws to reconstruct the defect of the iliac crest after harvesting. The result of this study revealed significant differences between the two groups at postoperative 1 week, 6 weeks and 3 months. No patient experienced chronic pain in the R group compared with the NR group. The results of other complications, cosmesis and satisfaction in the R group were better than those in the NR group.

Some researchers reconstructed the iliac crest after autografting using different methods and materials [10, 12]. Y-F Niu and colleagues shaped the allograft iliac crest to match the defect and reconstructed the anatomical contour, and then they fixed the allograft with hollow compression screws [10]. Jong Seong Lee and colleagues harvested a wedge of iliac crest with a bone burr on each wall and filled the defect with bone cement [12]. The results of theirs were similar in postoperative pain, cosmesis and additional complications. Both of them significantly reduced postoperative pain and yielded good cosmesis without increasing other adverse events.

Nonetheless, our method shows some improvements compared to the methods above. Bone cement is easier to obtain than matched iliac crest allografts which are difficult to acquire and carry exorbitant prices, often influencing some patients to refuse the reconstruction. Therefore, the surgery can be carried out in most hospitals. We were able to harvest autologous iliac crest grafts of different sizes and shapes because of the plasticity of bone cement. The surgical procedure is simple and generally takes less time. Compared to the research conducted by Jong Seong Lee et al., we added cancellous screws as a framework to provide an anchor for the bone cement and to prevent displacement and loosening; furthermore, bone cement is not limited to a particular shape.

The causes of postoperative pain after iliac crest harvesting are not clear, although we suspect that postoperative pain may be closely related to the following factors. The process of iliac crest harvesting causes damage to bone and soft tissue, and different sizes of autografts may lead to different degrees of pain [16]. The micromotion of the fracture, stimulation of the bone stump to the soft tissue, and adhesion and scarring of soft tissue are the main causes of postoperative pain. In our study, the decrease in pain may be related to the following factors. Firm fixation of the bone cement is the main reason, which can prevent micromotion of the fracture or between the bone cement and the walls of the bone defect. Bone cement reconstructs the anatomical shape of the iliac crest to reduce irritation to soft tissue. Careful separation of soft tissue and effective stitching can also prevent tissue adhesion and formation of scar.

Reconstruction of iliac crest donor site defects not only alleviates pain but also improves cosmesis and satisfaction. There was no collapse or displacement of implants, which could result in re-operation during the follow-up period, as reported by Jong Seong Lee et al. [12]. We believe that this method of reconstruction is more reliable which like a reinforced concrete structure, providing sufficient stability. In addition, this method could prevent some complications such as abdominal hernia theoretically.

This study still has some limitations. Although the results show that patients can benefit from reconstruction of the defect, the sample size was relatively small. Longer follow-up periods may discover additional differences, despite the reported advantages and disadvantages. More careful operation could avoid some complications, such as superficial numbness. In summary, a prospective randomized study with a larger sample and a longer follow-up are necessary. In addition, it should be considered whether pain is associated with the style of surgery that the patient underwent.

## Conclusions

Postoperative pain can be reduced and cosmesis can be improved through reconstructing the iliac crest defects after autogenous harvesting with bone cement and cancellous screws. The technique is simple, safe and easy to be implanted, making it possible to be performed in most hospitals. However, a larger sample of patients and a prospective, controlled study are necessary to verify and extend our results.

## Abbreviations

NR group: The not reconstructed group
pain VAS: The pain visual analog scale
R group: The reconstructed group
SSS: Surgical satisfaction scale
